# Draft Genome Sequence of *Saccharomyces cerevisiae* IR-2, a Useful Industrial Strain for Highly Efficient Production of Bioethanol

**DOI:** 10.1128/genomeA.01160-13

**Published:** 2014-01-16

**Authors:** Takehiko Sahara, Kazuhiro E. Fujimori, Maiko Nezuo, Masatoshi Tsukahara, Yuki Tochigi, Satoru Ohgiya, Yoichi Kamagata

**Affiliations:** Bioproduction Research Institute (BPRI), National Institute of Advanced Industrial Science and Technology (AIST), Tsukuba, Japana; BioJet Co., Ltd., Uruma, Japanb; Bioproduction Research Institute (BPRI), National Institute of Advanced Industrial Science and Technology (AIST), Toyohira, Sapporo, Japanc; Laboratory of Veterinary Physiology, Nippon Veterinary and Life Science University, Musashino, Tokyo, Japand

## Abstract

We sequenced the genome of *Saccharomyces cerevisiae* IR-2, which is a diploid industrial strain with flocculation activity and the ability to efficiently produce bioethanol. The approximately 11.4-Mb draft genome information provides useful insights into metabolic engineering for the production of bioethanol from biomass.

## GENOME ANNOUNCEMENT

*Saccharomyces cerevisiae*, the most popular ethanol-producing microorganism, has been used in the production of various alcoholic beverages. In the last few decades, a detailed understanding of the metabolic and genetic regulations of ethanol production from *S. cerevisiae* has become important for improved production of ethanol as a biofuel. Recently, several *S. cerevisiae* industrial strains have been sequenced (1–4). By comparing the publicly available genome sequences, a recent study revealed that *S. cerevisiae* species have a large number of genetic divergences (5). However, the genetic basis of their efficient bioethanol production remains unclear.

The flocculating *S. cerevisiae* strain IR-2 was originally isolated from a fermented food of Indonesia (6). Previous studies demonstrated that this strain has a high fermentation activity (7, 8). Further, the IR-2 strain, engineered with three enzyme genes for xylose metabolism (xylose reductase, xylitol dehydrogenase, and xylulose kinase), has been shown to efficiently produce ethanol from xylose (9, 10).

These data reveal that IR-2 potentially has a genetic background that is beneficial for effective bioethanol production from C_6_ and C_5_ carbon sources. A genomic analysis of IR-2 is therefore essential for further refinement of the xylose metabolic pathways and also provides a gateway for improving and developing effective systems for ethanol fermentation from different types of biomass.

Here, we report the draft genome sequence of the industrial diploid strain IR-2. The IR-2 genome was *de novo* sequenced with the GS FLX Titanium system (Roche Diagnostics, Switzerland) to highly oversample the genome (26.1-fold coverage), with a total of 956,160 reads and the generation of a paired-end library, enabling the assembly of 916 contigs into 90 supercontigs (scaffolds) using the GS *de novo* assembler software (Roche). A genome of 11.4 Mb was covered by 90 scaffolds (N_50_ scaffold length, 517,926 bases). Whole-genome resequencing analysis of the IR-2 genome was performed using the SOLiD 3 system (Life Technologies, Inc., Carlsbad, CA) to improve the sequence quality of the draft genome, and 251,770,705 50-base reads were obtained. The SOLiD 3 reads were aligned to the scaffolds by BWA (11), Bowtie (12), and SAMtools (13) to detect sequencing errors in the scaffolds. As a result, 3,606 nucleotide differences between the scaffolds generated by the GS FLX Titanium system and the reads generated by the SOLiD 3 system were revised. Five hundred ninety of the intercontig gaps were confirmed by PCR and closed by Sanger sequencing of the amplicons with the 3730xl DNA analyzer (Life Technologies).

Gene prediction and annotation were performed using Augustus software with the training set that is available for *S. cerevisiae* (14) and Exonerate software (15). The predicted proteins were searched against the curated open reading frames of the *Saccharomyces* Genome Database (SGD) (http://www.yeastgenome.org) (16) by BLASTp software (17), and matches were found for 5,628 protein-encoding genes at an *E* value cutoff of 10^−6^.

### Nucleotide sequence accession numbers.

The nucleotide sequences of the *S. cerevisiae* IR-2 draft genome have been deposited in DDBJ/EMBL/GenBank under the accession no. BAUI01000001 to BAUI01000322. The version described in this paper is the first version.
